# Impact of 1,7-malaria reactive community-based testing and response (1,7-mRCTR) approach on malaria prevalence in Tanzania

**DOI:** 10.1186/s40249-023-01166-0

**Published:** 2023-12-18

**Authors:** Wei Chang, Jessica Cohen, Duo-Quan Wang, Salim Abdulla, Muhidin Kassim Mahende, Tegemeo Gavana, Valerie Scott, Hajirani M. Msuya, Mary Mwanyika-Sando, Ritha John A. Njau, Shen-Ning Lu, Silas Temu, Honorati Masanja, Wilbald Anthony, Maru Aregawi W., Naveen Sunder, Tang Kun, Katia Bruxvoort, Jovin Kitau, Fadhila Kihwele, Godlove Chila, Mihayo Michael, Marcia Castro, Nicolas A. Menzies, Sein Kim, Xiao Ning, Xiao-Nong Zhou, Prosper Chaki, Yeromin P. Mlacha

**Affiliations:** 1 Department of Global Health and Population, Harvard T.H. Chan School of Public Health, Boston, MA USA; 2 Chinese Center for Disease Control and Prevention, National Institute of Parasitic Diseases, Shanghai, People’s Republic of China; 3 School of Global Health, Chinese Center for Tropical Diseases Research, Shanghai Jiao Tong University School of Medicine, Shanghai, People’s Republic of China; 4 Ifakara Health Institute, #5 Ifakara Street, Plot 463 Mikocheni, P.O. Box 78 373, Dar es Salaam, United Republic of Tanzania; 5 Africa Academy for Public Health, Dar es Salaam, Tanzania; 6 Muhimbili University of Health and Allied Sciences, Dar es Salaam, Tanzania; 7 Global Malaria Programme, World Health Organization, Geneva, Switzerland; 8 Bentley University, Waltham, MA USA; 9 Vanke School of Public Health, Tsinghua University, Beijing, People’s Republic of China; 10 School of Public Health, University of Alabama at Birmingham, Birmingham, AL USA; 11 The Pan-African Mosquito Control Association (PAMCA), KEMRI Headquarters, Mbagathi Road, Nairobi, 54840-00200 Kenya; 12 Chinese Center for Tropical Diseases Research, Shanghai, People’s Republic of China; 13 WHO Collaborating Centre for Tropical Diseases, Shanghai, People’s Republic of China; 14 National Center for International Research on Tropical Diseases, Ministry of Science and Technology, Shanghai, People’s Republic of China; 15 Key Laboratory of Parasite and Vector Biology, Ministry of Health, Shanghai, 200025 People’s Republic of China

**Keywords:** Malaria, Surveillance and response, Incidence rate, Community-health worker, Health facility, Community-based testing and treatment, 1,7-mRCTR, Tanzania

## Abstract

**Background:**

Progress in malaria control has stalled in recent years and innovative surveillance and response approaches are needed to accelerate malaria control and elimination efforts in endemic areas of Africa. Building on a previous China-UK-Tanzania pilot study on malaria control, this study aimed to assess the impact of the 1,7-malaria Reactive Community-Based Testing and Response (1,7-mRCTR) approach implemented over two years in three districts of Tanzania.

**Methods:**

The 1,7-mRCTR approach provides community-based malaria testing via rapid diagnostic tests and treatment in villages with the highest burden of malaria incidence based on surveillance data from health facilities. We used a difference-in-differences quasi-experimental design with linear probability models and two waves of cross-sectional household surveys to assess the impact of 1,7-mRCTR on malaria prevalence. We conducted sensitivity analyses to assess the robustness of our results, examined how intervention effects varied in subgroups, and explored alternative explanations for the observed results.

**Results:**

Between October 2019 and September 2021, 244,771 community-based malaria rapid tests were completed in intervention areas, and each intervention village received an average of 3.85 rounds of 1-7mRCTR. Malaria prevalence declined from 27.4% at baseline to 11.7% at endline in the intervention areas and from 26.0% to 16.0% in the control areas. 1,7-mRCTR was associated with a 4.5-percentage-point decrease in malaria prevalence (95% confidence interval: − 0.067, − 0.023), equivalent to a 17% reduction from the baseline. In Rufiji, a district characterized by lower prevalence and where larviciding was additionally provided, 1,7-mRCTR was associated with a 63.9% decline in malaria prevalence.

**Conclusions:**

The 1,7-mRCTR approach reduced malaria prevalence. Despite implementation interruptions due to the COVID-19 pandemic and supply chain challenges, the study provided novel evidence on the effectiveness of community-based reactive approaches in moderate- to high-endemicity areas and demonstrated the potential of South-South cooperation in tackling global health challenges.

**Graphical Abstract:**

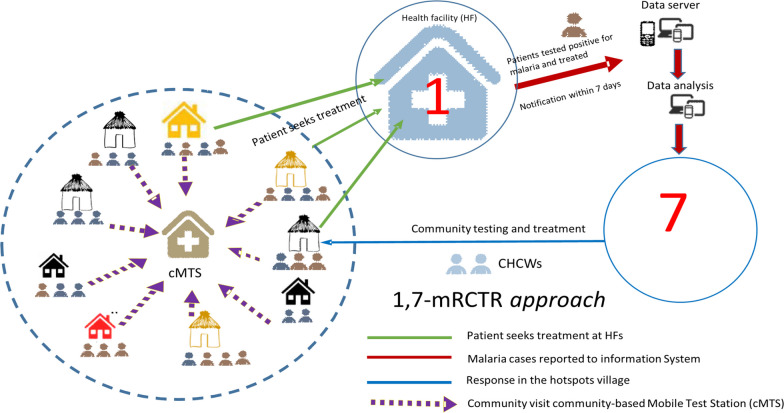

**Supplementary Information:**

The online version contains supplementary material available at 10.1186/s40249-023-01166-0.

## Background

Remarkable progress was achieved in reducing global malaria incidence and deaths between 2000 and 2015 [1]. More recently, this progress has plateaued and even reversed, with 247 million cases and 619,000 deaths in 2021 [2]. The vast majority of the global malaria burden (95%) remains concentrated in the WHO Africa Region [2]. High-transmission settings in sub-Saharan Africa have been affected by inadequate intervention coverage, gaps in health system access and quality, changes in vector bionomics, insecticide resistance, lack of rigorous routine surveillance systems, and poor socioeconomic conditions, including poor quality housing [2–4]. Health service disruptions during the COVID-19 pandemic have further strained health systems’ capacity to respond to malaria [5].

Strong surveillance and response systems are core to malaria elimination efforts, enabling targeted interventions based on high-quality data [6]. While malaria prevalence in Tanzania remains high overall, there is significant sub-national variation [7]. Strengthening the routine malaria surveillance system is essential to accurately map heterogeneous risks and better target interventions. Community-based surveillance and response strategies may support rapid detection and treatment of malaria infections and prevent onward transmission.

In low-endemic settings, active case detection has been used to screen and treat individuals at high risk of malaria infection [8–10]. Reactive case detection (RACD) is a specific active case detection strategy that involves reporting passively detected index cases, case investigation, and foci response to prevent further transmission. There is limited evidence demonstrating the effectiveness of RACD in interrupting malaria transmission; however, the strategy has been widely adopted in low-endemic settings in the Asia Pacific region and several countries in Africa, including Eswatini, South Africa, Namibia, Zambia, and Senegal [11–13]. As part of a successful effort to eliminate malaria by 2020, China implemented an adapted form of RACD called the “1–3–7” strategy [14, 15]. This model sets out timelines for each step of the RACD response: cases must be reported within one day, investigated within three days, and a foci response conducted within seven days. The “1–3–7” strategy has since been launched and adapted in several low-endemic countries, such as Cambodia [16] and Myanmar [17].

An adapted approach called the 1,7-malaria Reactive Community-based Testing and Response (1,7-mRCTR) approach was implemented in a China-UK-Tanzania project in the Rufiji district of Tanzania between 2015 and 2018 [18]. The 1,7**-**mRCTR approach relied on health facility data to identify villages with the highest malaria incidence and used community-based health care workers (CHCWs) to conduct screening and treatment of malaria infections in these villages. Due to the heavy logistical and operational demands of case-based surveillance and response, RACD strategies have typically been deployed only in settings with few malaria cases. The 1,7-mRCTR approach was the first attempt to pilot an adapted community-based reactive surveillance and response strategy in a high-endemic setting. The approach used health facility data to identify and target malaria hotspots and was associated with a substantial reduction in malaria burden in southern rural Tanzania. Further research was warranted given that the pilot study did not include an adequate control group and was conducted only in a single district, limiting the generalizability of the findings [18].

Building on the earlier pilot, this study aimed to evaluate the impact of the 1,7-mRCTR approach on malaria prevalence over two years in three districts of southeastern Tanzania using a control group. Study results may inform decision-making on whether and how the 1,7-mRCTR intervention should be scaled up under Tanzania’s National Malaria Control Program (NMCP). Our experience in this project also serves as a model for effective South-South cooperation to advance malaria elimination goals. To our knowledge, this is the first study of a scaled-up reactive community-based screen and treat strategy operating on 1–7 timeline targets in sub-Saharan Africa.

## Methods

### Study setting

This study was conducted between July 2019 and October 2021 in three districts in southeastern Tanzania: Kilwa district in Lindi region and Rufiji and Kibiti districts in Pwani region. The selection of these districts was purposive, taking into account logistical considerations and the variation in malaria prevalence as indicated by health facility data. In each district, two wards were selected representing four catchment populations. The catchment population in this context refers to the group of individuals who live within the service area of a particular health center. The boundaries of catchment areas are determined at the ward level—a local administrative division within a district—and determined based on health records. There was a total of 62 health facilities in the study area. These facilities included both public and private institutions, ranging from larger health centres which typically serve as the principal healthcare provider at the ward level to smaller dispensaries which are the primary healthcare units at the village level.

These catchment areas, including all villages located within these areas, were then assigned to either an intervention group or a control group based on malaria incidence rates (MIRs) and positivity rates. MIR is defined as the total number of malaria positive cases divided by population size from census conducted by the project team in 2019. Positivity rate is defined as the number of malaria positive cases divided by total number of malaria tests, recorded in the previous three years at the health facility level. In this study, the primary operational level was a village with an average of 2500–5500 inhabitants. We also imposed a minimum distance of 30 km between the centers of intervention and control wards to minimize spillover. Based on the 2012 census, the total population for these selected wards was approximately 243,449 people.

### 1,7-mRCTR approach

The 1,7-mRCTR approach involved reporting any confirmed malaria cases at health facilities within one day and conducting follow-up community-wide testing in selected villages within seven days to slow malaria transmission in the same phase of the *Plasmodium* life cycle. The main intervention was community-based malaria testing and treatment based on surveillance data from health facilities. We developed a case-based reporting system using the Open Data Kit (https://opendatakit.org/) tool to capture information on malaria cases at health facilities. The reporting system was compatible with the District Health Information Software 2 (DHIS2) platform (https://www.dhis2symposium.org/) and allowed data aggregation and sharing. We provided tablets to health facilities to collect case-based data, including patients’ demographic information and their residence village. Data from all confirmed malaria cases were aggregated weekly to calculate a village-level MIR.

Villages with the highest MIR within each catchment area were targeted for community-based malaria testing and treatment in each week. Field supervisors first notified local leaders that their villages were identified as malaria hotspots for malaria testing and treatment. Local leaders then met with CHCWs, who were recruited from the study areas, to select the location for community mobile testing stations. The testing stations were placed in areas with clustered households to provide easy access for community members. From Monday to Friday, CHCWs set up testing stations in different hamlets of the targeted villages, starting with those presumed to have the highest burden of malaria cases and moving around to increase testing coverage. If there was a school in the targeted village, the testing and treatment campaign was conducted on the premises of the school at least one day a week. The field team requested head teachers to provide a list of students who lived in the targeted village, and while other students were invited to participate in testing and treatment campaigns, only data from students residing in targeted villages were recorded.

All individuals who were at least six months of age were eligible to participate in malaria testing and treatment. Malaria testing was done with rapid diagnostic tests [RDTs; CareStartTM Malaria Pf/PAN (HRP2/pLDH) Ag Combo, Access Bio, Inc 65 Clyde Rd., Suite A, Somerset, NJ 08873, USA] at community testing stations. Participants who tested positive were given a short survey, including questions about their travel and medical history, and offered a full regiment of artemisinin-based combination therapies (dihydroartemisinin piperaquine phosphate) according to the National Malaria Treatment Guidelines [7]. The first dose of malaria treatment was given via directly observed treatment at the testing station. Pregnant women who tested positive for malaria were referred to nearby health facilities for follow-up care.

A social team comprised of sociologists, CHCWs, and local leaders conducted a series of activities before and during the campaigns to encourage community participation and offer health education. In the week preceding the campaigns, the social team sent out messages via megaphones to encourage voluntary malaria testing and emphasized that testing and treatment were provided at no cost. The social team also distributed user-friendly booklets and posters that were written in the local language and highlighted the importance of early testing, treatment adherence, use of long-lasting insecticidal nets, and environmental control. Detailed activities for the 1,7-mRCTR intervention have been published previously [18, 19].

In addition to malaria testing and treatment, larviciding was provided in the intervention wards in Rufiji district to reduce the vector population and to further drive malaria transmission towards a pre-elimination phase. Larviciding was implemented between November 2020 and October 2021 through a community-based approach using either *Bacillus thuringiensis var. israelensis* or *Bacillus sphaericus*, called BACTIVEC and GRISELESF, respectively, and produced in Tanzania. All the larval habitats identified in the area were targeted with larvicide application weekly, except when interrupted by either heavy rains or floods. Villages in both the intervention and control areas continued to benefit from the routine malaria control program implemented by the National Malaria Control Programme, such as vector control, distribution of long-lasting insecticide-treated nets, and routine malaria case management at health facilities.

### Data collection via household surveys

We used two waves of cross-sectional household surveys to assess the effects of the 1,7-mRCTR approach in the study wards located in three districts of Tanzania. The baseline survey was conducted between July 24, 2019 and September 4, 2019, and the endline survey was conducted between September 20, 2021 and October 27, 2021. We used a stratified sampling approach to select households within a village and then individuals within a household. All households located in the study ward were enumerated and a random sample of households was recruited to participate in the study. If a household was not available for the interview, we recruited the household next door.

Within each household, we first interviewed the head of household and then randomly selected one available household member from each of three age groups (under 5 years, between 5 and 15 years, and above 15 years) to participate in the survey. For individuals aged 15 or under, we obtained informed assents and parental consents before conducting interviews and malaria tests. For children who were unable to respond to the survey themselves, we interviewed the head of household or a child’s caregiver on questions related to the use of preventive measures and care seeking behaviors.

The household surveys were developed based on the Malaria Indicator Survey Tool and collected data on socio-economic characteristics, knowledge, and use of malaria preventative measures, health expenditures, use of health services, and travel history [20]. We also collected blood samples from selected household members for malaria testing, including RDTs, blood smears to determine parasite density at a central laboratory, and dried blood spots collected on filter paper and preserved for later PCR-based parasite detection. All blood samples were drawn from a single finger prick to collect a total of ≤ 30 μl of blood. Participants who tested positive based on RDT results were offered free treatment according to the National Malaria Treatment Guidelines, and the first dose was given via directly observed therapy. If a participant refused this treatment option, they were referred to a nearby health facility with logistical assistance.

### Outcomes

The main outcome was malaria status assessed via RDTs. The secondary outcomes included self-reported fever in the previous 14 days and underarm temperature measured using a digital thermometer during household surveys. To determine sample size, we used a stratified sampling approach to select the number of villages and then the number of households within each village [21], using malaria prevalence data from health facilities and population data from the National Bureau of Statistics which is public available (www.nbs.go.tz). The precision of the estimated sample size for areas with low parasitemia was set to 0.03 to adjust for the difference in prevalence between community and health facility estimates. We assumed a non-response rate of 10% and an average of five residents per household. Weights calculated as the relative proportion of a village population size to the total stratum population were applied to obtain the final sample size.

### Ethical considerations

Informed consent was obtained from heads of household and household members who were 18 years of age or above. For those under 18 years of age, informed consent was obtained from parents or guardians. The consent forms were prepared in English and translated into Kiswahili. For individuals who were not able to read, the informed consent form was read out by the local CHCWs in the presence of a community witness, and the participant was asked to mark a thumb impression on the form. Institution ethical approval was obtained from the Ifakara Health Institute Institutional Review Board (IHI/IRB/EXT/No: 18–2020) and the National Institute of Medical Research (NIMR/HQ/R.8a/Vol. IX/3634).

### Statistical analysis

We used a difference-in-differences (DID) quasi-experimental design to assess the impact of the 1,7-mRCTR approach on malaria prevalence. The DID design compares changes in malaria prevalence before and after the intervention in “treatment” areas, with changes in malaria prevalence during the same period in “control” areas. A key assumption of the DID methodology is that changes in outcomes in the treatment areas would have been similar to the changes in outcomes in the control areas in the absence of the intervention [22].

We analyzed outcomes using a multi-level regression model, in which individuals are nested within households which are nested within villages. The primary outcome in the linear probability model was a binary variable equal to one if an individual tested positive for malaria. The dependent variables included a binary “post” variable equal to one for the endline survey measures and zero for the baseline survey measures, an interaction between the “post” variable and an indicator variable equal to one if the individual lived in a treatment village, and a full set of village fixed effects. The model controlled for household characteristics (ownership of any treated mosquito net, flush toilet, improved source of drinking water, house ownership, and health insurance) and household member characteristics (age and sex). Standard errors were adjusted for clustering at the household level to account for the correlation of members from the same households.

We conducted several sensitivity analyses to assess the robustness of our results. First, we compared the results from alternative model specifications, including probit models instead of linear probability models, clustering standard errors at the village and ward levels instead of the household level, and including different sets of control variables. Second, since treatment assignment was not random, we performed coarsened exact matching on average age and access to improved water sources, two variables that were significantly different at baseline, at the village level to improve the comparability between the intervention and comparison groups [23]. Specifically, we used baseline data aggregated at the village level to match villages and then compared endline malaria prevalence among matched villages, applying weights generated from the baseline data. This matching procedure retained 55 out of the 85 villages that were surveyed in both waves and achieved a better balance in village characteristics in the baseline (Additional file 1: Table S1). We only included two variables because adding a third matching variable retained fewer than one third of the villages in the original sample.

Since intervention areas in Rufiji district received larviciding treatment in addition to the other components of the intervention, we assessed the intervention effects in Rufiji separately in addition to the overall effects in the three districts combined. We conducted subgroup analyses to assess intervention effects by age, sex, education, and household wealth. We also assessed whether the effects of the intervention varied by treatment intensity, defined by the number of testing and treatment campaigns a village received during the intervention period. These regression models included an additional triple-interaction term of survey round, intervention arm, and number of treatment rounds. In alternative models, we defined treatment intensity as a binary variable and considered a village to be “highly treated” if it had more than the median number of treatment rounds.

To explore alternative explanations for declines in malaria prevalence besides the intervention, we assessed changes in malaria-related knowledge and travel history. To test whether differences in household characteristics between intervention and control areas could cause confounding, we also examined whether there was any association between the intervention arm, survey round, and key household-level characteristics. Since the baseline and endline surveys were conducted in different months of the year, we used daily agro-climatology data produced through the Prediction of Worldwide Energy Resources project to examine changes in climate patterns in the study areas, including temperature, precipitation, humidity, and surface soil wetness [24]. All analyses were performed using Stata version 15 (StataCorp, College Station, USA).

## Results

### Impact of 1,7-mRCTR on the reduction of malaria prevalence

Overall, 11,655 participants from 5757 households completed the baseline survey and 12,660 participants from 5472 households completed the endline survey. Participation refusal in the surveys was lower than 1%. At baseline, 28.6% of the households were female-headed and 61.5% of the surveyed household members were female (Table 1). About half of the household members were 15 years of age or below. The majority of surveyed household members (87.1%) reported having slept under a mosquito net in the previous night and 11.4% reported having had a fever in the previous 14 days. According to the RDT results at baseline, 27.4% of those from the intervention villages tested positive for malaria compared to 26.0% in control villages.

**Table 1:** Characteristics of participants, households, and villages in the baseline surveys

	All	Control	Intervention
*Panel A: Household characteristics*			
Number of households	5757	2581	3176
Female household head*, n* (%)	1646 (28.6%)	709 (27.5%)	937 (29.5%)
Household head completed primary school, *n* (%)	3763 (65.4%)	1776 (68.8%)	1987 (62.6%)
Owns house,* n* (%)	4994 (86.7%)	2330 (90.3%)	2664 (83.9%)
Land, *n* (SD)	3.87 (5.71)	4.18 (6.16)	3.62 (5.32)
Improved source of drinking water,* n* (%)	4747 (82.5%)	2008 (77.8%)	2739 (86.2%)
Flush toilet,* n* (%)	387 (6.7%)	114 (4.4%)	273 (8.6%)
Health insurance, *n* (%)	618 (10.7%)	240 (9.3%)	378 (11.9%)
Has any treated mosquito nets, *n* (%)	3456 (60.0%)	1682 (65.2%)	1774 (55.9%)
Agriculture as main income source*, n* (%)	4709 (81.8%)	2236 (86.6%)	2473 (77.9%)
Number of household members, mean (SD)	4.82 (2.49)	4.45 (2.50)	5.11 (2.44)
*Panel B: Characteristics of household members surveyed*			
Number of individuals	11,655	4,927	6,728
Female, *n* (%)	7161 (61.4%)	3034 (61.6%)	4127 (61.3%)
Age category (years)			
Less than 5, *n* (%)	2578 (22.1%)	1009 (20.5%)	1569 (23.3%)
5 to 15, *n* (%)	3283 (28.2%)	1310 (26.6%)	1973 (29.3%)
Above 15, *n* (%)	5794 (49.7%)	2608 (52.9%)	3186 (47.4%)
Had fever in the past 14 days, *n* (%)	1332 (11.4%)	503 (10.2%)	829 (12.3%)
Knows what malaria is, *n* (%)	5481 (74.5%)	2410 (74.1%)	3071 (74.8%)
Number of malaria symptoms listed (max = 4), *n* (SD)	1.96 (0.87)	1.84 (0.87)	2.05 (0.86)
Number of malaria prevention measures listed (max = 3), *n* (SD)	1.48 (0.71)	1.41 (0.66)	1.54 (0.74)
Slept under a mosquito net last night, *n* (%)	10,151 (87.1%)	4235 (86.0%)	5916 (87.9%)
Weight-for-age Z-score for those under 5, *n* (SD)	-0.56 (1.81)	-0.56 (1.92)	-0.57 (1.74)
Body temperature, degrees Celsius, *n* (SD)	36.77 (0.44)	36.72 (0.40)	36.81 (0.46)
Malaria rapid diagnostic test—positive result, *n* (%)	3123 (26.8%)	1279 (26.0%)	1844 (27.4%)
*Panel C: Village-level characteristics*			
Number of villages	86	27	59
Population from census, *n* (SD)	2105 (1471)	1469 (909)	2429 (1599)
Number of households interviewed, *n* (SD)	66.94 (59.20)	95.59 (71.53)	53.83 (47.81)

*Agricultural source of income* includes farming, fishing, or livestock keeping

*Has any treated mosquito nets* is coded to 1 if the answers to “Does your household have any mosquito nets?” and “Are/Is your net(s) treated (LLIN)?” were both Yes

Questions on malaria knowledge were only asked to respondents above 5 years of age. *Number of malaria symptoms listed* is based on how many of the following four symptoms a respondent could name without prompt: chills, fever, cold, and headache. *Number of malaria prevention measures listed* is based on how many of the following three measures a respondent could name without prompt environmental hygiene and cleanliness, mosquito net use, and mosquito repellent/incense

*Slept under a mosquito net last night* is coded to 1 if the answer to “Did you sleep under a net last night?” was Yes and coded to 0 if the answer is No, Not Applicable, or missing

Numbers of villages at baseline and endline are different due to changes in administrative boundaries

**Fig. 1.**
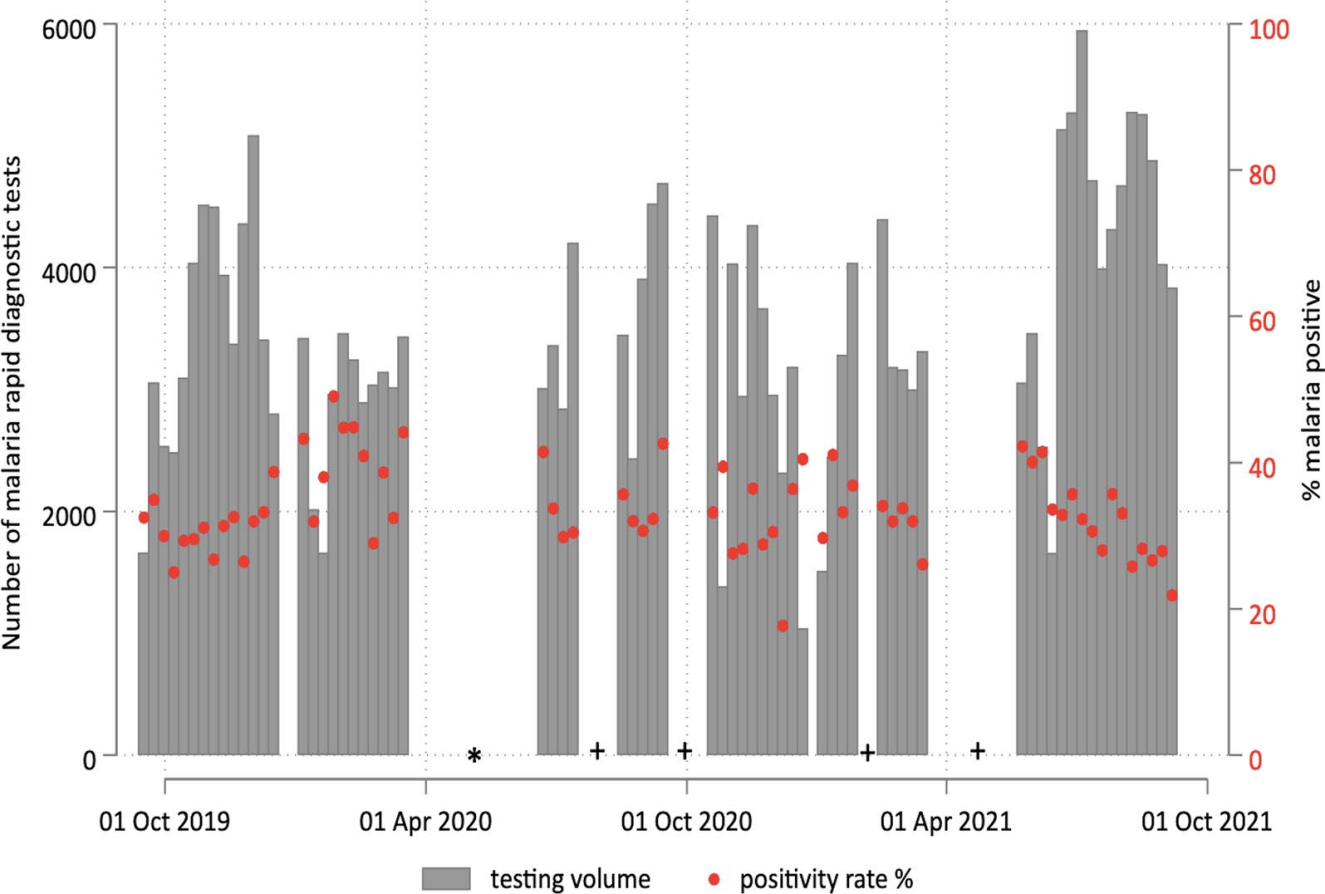
1,7-mRCTR testing volume and positivity rate by week. *Interruption due to the COVID-19 pandemic; ^+^ Interruption due to stockout of rapid diagnostic tests. *1,7-mRCTR* 1,7-malaria Reactive Community-Based Testing and Response

**Fig. 2:**
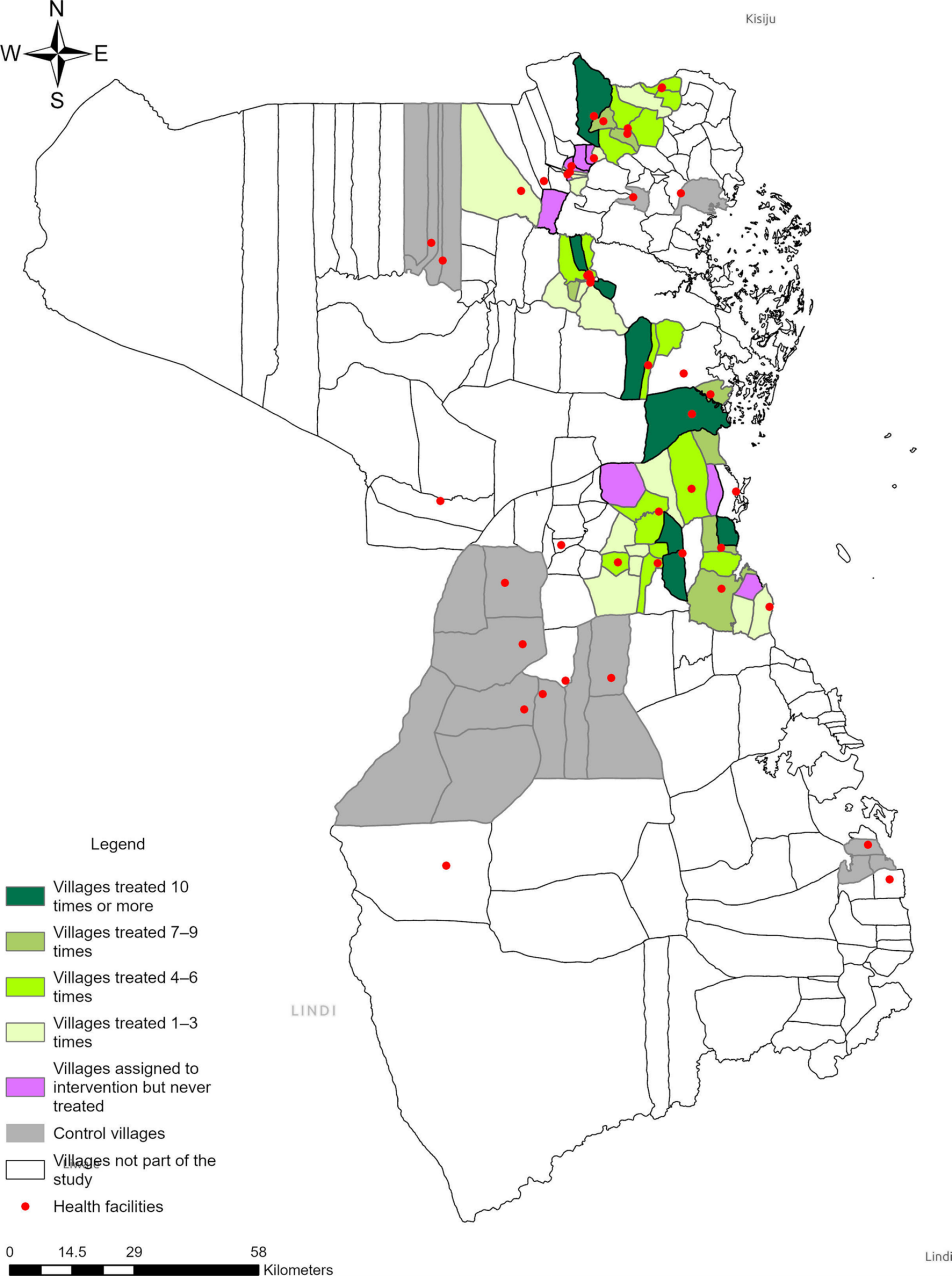
Map of study villages and health facilities

SD: standard deviation;

Between October 2019 and September 2021, a total of 244,771 malaria RDTs were completed in the intervention villages through testing and treatment campaigns (Table 2). The intervention was paused for 13 weeks due to the COVID-19 pandemic and another 19 weeks due to stockouts of RDTs (Fig. 1). The average age of campaign participants was 19.3 years and 54.1% were female. Based on RDTs conducted as part of the intervention, 33.8% of the hotspot villages tested positive for malaria and the positivity rate stayed flat between 2019 and 2021 (Table 2 and Fig. 1). Villages in the intervention group received an average of 3.85 rounds of 1,7-mRCTR, among which seven villages did not receive any test and treat campaign and nine villages received at least ten rounds of campaigns (Fig. 2).

**Table 2:** 1,7-mRCTR campaigns: testing volume and participant characteristics

	All years	2019	2020	2021
*All districts*				
Number of tests	244,771	48,870	98,413	97,488
Age, years, *n* (SD)	19.34 (16.95)	19.34 (17.40)	19.45 (17.17)	19.24 (16.50)
Female, *n* (%)	132,357 (54.1%)	26,649 (54.5%)	53,525 (54.4%)	52,183 (53.5%)
Pregnant, *n* (%)	756 (0.6%)	209 (0.8%)	373 (0.7%)	174 (0.3%)
Body temperature, degrees Celsius, *n* (SD)	36.53 (0.35)	36.63 (0.39)	36.58 (0.34)	36.44 (0.31)
Positive RDT result, *n* (%)	82,742 (33.8%)	15,067 (30.8%)	35,939 (36.5%)	31,736 (32.6%)
Travelled in the past 14 days, *n* (%)	721 (0.9%)	316 (2.1%)	340 (0.9%)	65 (0.2%)
Travelled to rural area, *n* (%)	379 (52.6%)	173 (54.7%)	175 (51.5%)	31 (48.0%)
Had fever in the past 14 days, *n* (%)	11,398 (13.8%)	1887 (12.5%)	4599 (12.8%)	4912 (15.5%)
*Rufiji*				
Number of tests	93,660	16,532	36,454	40,674
Age, years, *n* (SD)	19.00 (16.67)	19.46 (17.62)	19.16 (16.89)	18.66 (16.05)
Female, *n* (%)	51,604 (55.1%)	9264 (56.0%)	19,950 (54.7%)	22,390 (55.0%)
Pregnant, *n* (%)	386 (0.7%)	122 (1.3%)	175 (0.9%)	89 (0.4%)
Body temperature, degrees Celsius, *n* (SD)	36.53 (0.32)	36.64 (0.37)	36.59 (0.31)	36.44 (0.29)
Positive RDT result, *n* (%)	16,082 (17.2%)	2,370 (14.3%)	7,201 (19.8%)	6,511 (16.0%)
Travelled in the past 14 days, *n* (%)	189 (1.2%)	48 (2.0%)	100 (1.4%)	41 (0.6%)
Travelled to rural area, *n* (%)	119 (63.0%)	31 (65.0%)	67 (67.0%)	21 (51.0%)
Had fever in the past 14 days, *n* (%)	1457 (9.1%)	186 (7.8%)	683 (9.5%)	588 (9.0%)

Travel history and fever were only asked among those who tested positive

Testing coverage is calculated by dividing the total number of RDT per village-week by village population from the census, using targeted villages, and keeping only village-weeks with more than 100 RDT tests done

*1,7-mRCTR* 1,7-malaria Reactive Community-Based Testing and Response; *SD* standard deviation; *RDT* rapid diagnostic test

^*^District is based on the location of testing stations in targeted villages

**Fig. 3:**
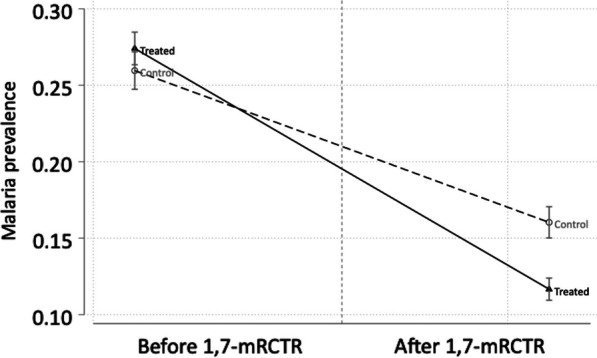
Changes in malaria prevalence before and after the implementation of the 1,7-mRCTR approach. Malaria prevalence defined as fraction tested malaria positive with malaria rapid diagnostic tests. *1,7-mRCTR* 1,7-malaria Reactive Community-Based Testing and Response

Malaria prevalence declined from 27.4% at baseline to 11.7% at endline in the intervention arm and from 26.0% to 16.0% in the control arm, based on RDT results from household surveys (Fig. 3). Regression estimates from the DID model suggest that the intervention was associated with a 4.5-percentage-point reduction in malaria prevalence [95% confidence interval (*CI*): -0.067, -0.023], which is equivalent to a 17% reduction from the baseline malaria prevalence of 26.0% in the comparison villages (Table 3). In Rufiji, the district where larviciding was also part of the intervention, 1,7-mRCTR was associated with a 4.6-percentage-point reduction in malaria prevalence (95% *CI:* − 0.081, − 0.011), representing a decline of 63.9% from the baseline malaria prevalence of 7.2% in the comparison villages of Rufiji.

**Table 3:** Effects of the 1,7-mRCTR approach on malaria prevalence and fever

	(1)	(2)
	All districts	Rufiji
*Positive malaria test result, all age groups*		
Difference-in-differences coefficient estimate	− 0.045^***^	− 0.046^**^
	[− 0.067, − 0.023]	[− 0.081, − 0.011]
Change in comparison group	− 0.122^***^	− 0.014
	[− 0.139, − 0.104]	[− 0.045, 0.017]
Observations	24,102	4926
Adjusted *R*^2^	0.1537	0.0367
Mean of comparison group at baseline	0.260	0.072
Villages	88	22
*Self-reported fever in past 14 days, under five years of age*		
Difference-in-differences coefficient estimate	-0.047**	0.032
	[− 0.082, − 0.011]	[− 0.046, 0.110]
Change in comparison group	− 0.089***	− 0.098**
	[− 0.117, − 0.061]	[− 0.163, − 0.033]
Observations	5308	1092
Adjusted *R*^2^	0.0518	0.0489
Mean of comparison group at baseline	0.153	0.133
Villages	88	22
*Body temperature of 38 ℃ or above, under 5 years of age*		
Difference-in-differences coefficient estimate	− 0.003	− 0.005
	[− 0.010, 0.005]	[− 0.013, 0.004]
Change in comparison group	− 0.004	− 0.000
	[− 0.010, 0.001]	[− 0.003, 0.003]
Observations	5271	1082
Adjusted *R*^2^	0.0011	0.0064
Mean of comparison group at baseline	0.006	0.000
Villages	88	22

95% confidence intervals in brackets

Linear probability models

Included village fixed effects and controlled for household characteristics (mosquito nets, flush toilet, improved source of drinking water, house ownership, health insurance) and individual member characteristics (age and sex)

The number of villages is 86 at baseline (instead of 88 as shown here) because of changes in administrative boundaries

Standard errors clustered at the household level

Data at household member level

*1,7-mRCTR* 1,7-malaria Reactive Community-Based Testing and Response

^*^P < 0.05, ^**^P < 0.01, ^***^P < 0.001

### Sensitivity analysis

The effects of the intervention on malaria prevalence were comparable in probit models (Additional file 1: Table S2) and in models with varying sets of control variables (S3 Table). Clustering standard errors at the village level (95% *CI:* − 0.100, 0.009) produced statistically insignificant results (Additional file 1: Table S4). Coarsened exact matching produced similar results when using the full sample of all three districts (Table 4): among villages retained after matching, the intervention was associated with a 3.6-percentage-point reduction (95% *CI:* − 0.059, − 0.014) in malaria prevalence in the unweighted model and a 5.8-percentage-point reduction (95% *CI:* − 0.080, − 0.037) in the weighted model. However, the effects in Rufiji were no longer statistically significant after matching (Table 4).

**Table 4:** Effects of the 1,7-mRCTR approach on malaria prevalence with matching

	(1)	(2)
	All districts	Rufiji
Panel A: full sample		
Difference-in-differences coefficient estimate	− 0.051^***^	− 0.050^***^
	[− 0.071, − 0.032]	[− 0.080, − 0.021]
Change in comparison group	− 0.103^***^	− 0.004
	[− 0.118, − 0.088]	[− 0.029, 0.020]
Observations	24,044	4926
Adjusted *R*^2^	0.1134	0.0260
Mean of comparison group at baseline	0.260	0.072
Villages	85	22
Panel B: Villages retained after coarsened exact matching, unweighted		
Difference-in-differences coefficient estimate	− 0.036^**^	− 0.017
	[− 0.059, − 0.014]	[− 0.051, 0.018]
Change in comparison group	− 0.116^***^	− 0.034^*^
	[− 0.131, − 0.100]	[− 0.062, − 0.005]
Observations	17,708	3111
Adjusted *R*^2^	0.1200	0.0292
Mean of comparison group at baseline	0.268	0.078
Villages	55	13
Panel C: Villages retained after coarsened exact matching, weighted		
Difference-in-differences coefficient estimate	− 0.058^***^	− 0.018
	[− 0.080, − 0.037]	[− 0.057, 0.020]
Change in comparison group	− 0.094^***^	− 0.032
	[− 0.109, − 0.079]	[− 0.065, 0.001]
Observations	18,730	2817
Adjusted *R*^2^	0.1107	0.0323
Mean of comparison group at baseline	0.268	0.078
Villages	55	13

95% confidence intervals in brackets

Linear probability models

Included village fixed effects and controlled for household characteristics (mosquito nets, flush toilet, improved source of drinking water, house ownership, health insurance) and individual member characteristics (age and sex)

In A, we used a subsample of villages that were in both baseline and endline household surveys. In B and C, we used a subsample of villages retained after Coarsened Exact Matching. In C, we applied weights generated from Coarsened Exact Matching using village-level data matched on age and source of drinking water

Standard errors clustered at the household level

Data at household member level

*1,7-mRCTR* 1,7-malaria Reactive Community-Based Testing and Response

^*^P < 0.05, ^**^P < 0.01, ^***^P < 0.001

**Fig. 4:**
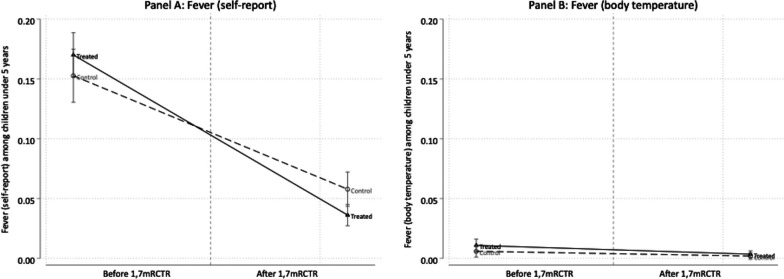
Changes in fever among children under 5 years of age before and after the implementation of the 1,7-mRCTR approach. Fever (self-report) is defined as fraction responded yes to a survey question that asked about fever in past 14 days. Fever (body temperature) is defined as fraction with body temperature of 38 ℃ or above. *1,7-mRCTR* 1,7-malarla Reactive Community-Based Testing and Response

Self-reported fever in the previous 14 days among children under 5 years of age also declined from 16.3% in the baseline to 4.4% in the endline, while fever based on body temperature measured at the time of the household survey was rare in both waves (Fig. 4). The DID model suggested that the intervention was associated with a 4.7-percentage-point reduction in self-reported fever among children under 5 years of age (95% *CI:* − 0.082, − 0.011), representing a 30.7% reduction from the baseline level of 15.3% in the comparison group (Table 3). The intervention was not significantly associated with any change in fever based on body temperature or change in fever in the Rufiji-specific models among those under 5 years of age.

### Effects of 1,7-mRCTR in subgroups

**Fig. 5:**
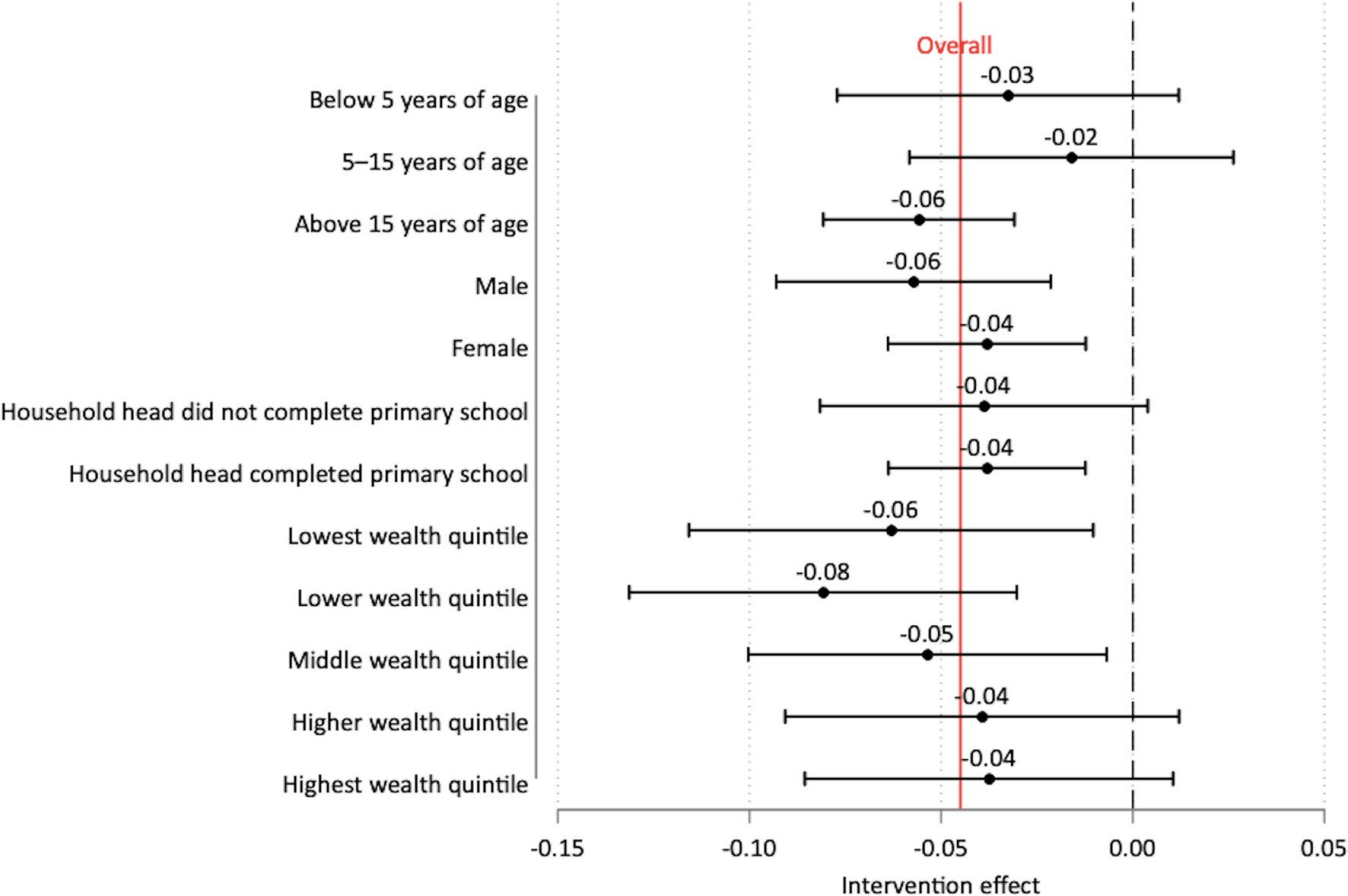
Effects of the 1,7-mRCTR approach on malaria prevalence in key subgroups. Linear models that show changes in percentage point (coefficients from difference-in-differences models) and 95% confidence intervals. Included village fixed effects and controlled for household characteristics (mosquito nets, flush toilet, improved source of drinking water, house ownership, health insurance) and individual member characteristics (age and/or sex). Standard errors clustered at household level. *1,7-mRCTR* 1,7-malaria Reactive Community-Based Testing and Response

The effects of the intervention in the subgroups of interest are shown in Fig. 5 and Additional file 1: Tables S5–8. The intervention was associated with a 5.6-percentage-point reduction (95% *CI:* − 0.081, − 0.031) in malaria prevalence among individuals that were above 15 years of age (Additional file 1: Table S5) but not younger age groups in any district. The intervention was also associated with a 3.8-percentage-point reduction (95% *CI:* − 0.064, − 0.012) in malaria prevalence among individuals from households whose heads had completed primary school (Additional file 1: Table S7). Regarding economic background, the intervention was associated with a 6.3-percentage-point (95% *CI:* − 0.116, − 0.010), 8.1-percentage-point (95% *CI:* − 0.131, − 0.030), and 5.4-percentage-point (95% *CI:* − 0.100, − 0.007) decreases in malaria prevalence among individuals from households in the lowest, lower, and middle wealth index quintiles respectively (Additional file 1: Table S8).

### Effects of 1,7-mRCTR by treatment intensity

The effects of the intervention by treatment intensity are shown in Table 5. For villages in the interventionarm, neither one additional round of treatment nor receiving more than four rounds of treatment (the median number of treatment rounds for all intervention villages) was associated with a greater decline in malaria prevalence compared with the intervention villages that received less intensive treatment.

**Table 5:** Effects of the 1,7-mRCTR approach by treatment intensity

	(1)	(2)
	All districts	Rufiji
*Panel A: one additional treatment round*		
Intervention # Endline # Treatment round	− 0.001	− 0.004
	[− 0.004, 0.002]	[− 0.009, 0.000]
Intervention # Endline	− 0.040^**^	− 0.018
	[− 0.067, − 0.013]	[− 0.061, 0.025]
Change in comparison group	− 0.122^***^	− 0.014
	[− 0.139, − 0.104]	[− 0.045, 0.017]
Observations	24,102	4926
Adjusted *R*^2^	0.1534	0.0377
Mean of comparison group at baseline	0.267	0.081
*Panel B: High treatment intensity*		
Intervention # Endline # Highly treated	0.024	− 0.002
	[− 0.004, 0.051]	[− 0.036, 0.033]
Intervention # Endline	− 0.056^***^	− 0.045^*^
	[− 0.082, − 0.031]	[− 0.085, − 0.005]
Change in comparison group	− 0.122^***^	− 0.014
	[− 0.139, − 0.104]	[− 0.045, 0.017]
Observations	24,102	4926
Adjusted *R*^2^	0.1538	0.0365
Mean of comparison group at baseline	0.260	0.072

95% confidence intervals in brackets

A village is defined as highly treated if it was treated more than four times (the median of intervention villages)

Included village fixed effects and controlled for household characteristics (mosquito nets, flush toilet, improved source of drinking water, house ownership, health insurance) and individual member characteristics (age and sex)

Standard errors clustered at the household level

Data at household member level

*1,7-mRCTR* 1,7-malaria Reactive Community-Based Testing and Response

^*^P < 0.05, ^**^P < 0.01, ^***^P < 0.001

### Alternative explanations for changes in malaria prevalence

Considering other factors that might have affected malaria prevalence, knowledge of malaria improved from baseline to endline in both the intervention and comparison villages, and the intervention did not have a statistically significant association with malaria knowledge (Additional file 1: Table S9). Meanwhile, travel outside of the village fell by 3.5 percentage points (95% *CI:* − 0.044, − 0.026) from 5.6% in the comparison villages in the baseline and declined by an additional 2.0 percentage points in the intervention group (95% *CI:* − 0.031, − 0.008). Changes in household characteristics from baseline to endline did not differ by intervention arm (Additional file 1: Table S10). Temperature and precipitation patterns did not change between the months of the baseline survey and the months of the endline survey in the study areas (Additional file 1: Fig. S11).

## Discussion

This study used a quasi-experimental design to assess the impact of the 1,7-mRCTR approach implemented at scale over two years in three districts of Tanzania. In these districts we found that the 1,7-mRCTR intervention reduced malaria prevalence in intervention areas as compared to matched control areas, and these findings were robust to alternative analytic assumptions. Despite the implementation interruptions due to the COVID-19 pandemic and the related challenges in the RDT supply chain and healthcare delivery, the study provides novel evidence on the effectiveness of reactive surveillance approaches in moderate- to high-endemic areas and confirms the operational feasibility of a community-based, reactive screen and treat program using surveillance data.

The 1,7-mRCTR approach was associated with a 17% reduction in malaria prevalence in three districts in Tanzania characterized by moderate- to high-malaria transmission. This result is similar to the findings from a randomized controlled trial of three rounds of a mass screening and treatment (MSAT) intervention in Zambia where malaria prevalence in intervention and control areas was similar pre-intervention (34.5% and 38.5%, respectively), but declined to 29.2% in intervention areas and rose to 44.0% in control areas [25]. In the Zambia study, total outpatient malaria cases declined by 17% more in the intervention areas as compared to control areas. In contrast, a randomized controlled trial from Burkina Faso found that an MSAT intervention for asymptomatic malaria infections was not effective in reducing symptomatic malaria incidence [26]. In general, evidence from MSAT programs in moderate to high transmission settings has been mixed in terms of effectiveness in reducing malaria transmission [27]. Together with findings from our study, the limited and mixed evidence calls for future research to innovate and test novel MSAT approaches in moderate- to high-malaria transmission settings as countries mobilize resources and galvanize increased health system capacity for malaria elimination.

Our finding that the largest declines in malaria prevalence (63.9% decline) were seen in Rufiji, a low-endemic district, suggests that the 1,7-mRCTR approach may be most effective when used in lower transmission areas and combined with larviciding. Although community-based testing and treatment make 1,7-mRCTR different from the traditional RACD approaches, this result is consistent with previous evidence from the Asia Pacific region and several countries in Africa that showed the effectiveness of reactive approaches in low-endemic settings [11–13]. It also echoed the WHO guidelines that recommend adding supplementary programming, such as larviciding, to the implementation of surveillance strategies [6].

The 1,7-mRCTR intervention had the largest impact among households from lower wealth index quintiles, suggesting the potential of community-based approaches to improve the access and utilization of malaria testing and treatment among the most disadvantaged populations. There is strong evidence that the uptake of malaria preventive measures and quality treatment is closely related to socioeconomic status, as individuals with low socio-economic status have limited financial resources, time, and health literacy, among other barriers to good quality of care [4, 28–30]. Since achieving equity in the distribution of health services and interventions is a key principle of Tanzania’s National Malaria Strategic Plan, the 1,7-mRCTR intervention should be considered an effective and equitable approach in the efforts toward malaria elimination [7].

Similarly to the pilot study, we observed a substantial decline in malaria prevalence in the control areas, from 26.0% to 12.2% over the two years. In contrast to the pilot study, the baseline and endline household surveys were conducted around the same time of the year and the control areas did not receive any additional programming other than the routine malaria prevention and control program implemented by the NMCP. This result was robust in alternative model specifications, and our climate data did not indicate any difference in the climate factors that may affect malaria transmission. One explanation could be the improvement of malaria prevention knowledge thanks to other ongoing health education led by the NMCP, as individuals in the control areas had greater knowledge of malaria, its associated symptoms, and prevention measures in the endline. Another explanation might be declining travel between high- and low-endemic areas as a result of the COVID-19 pandemic. While the large effect size could not be explained by increased knowledge or decreased travel alone, pinpointing other factors that contributed to this substantial change in malaria prevalence independent of the 1,7-mRCTR intervention would be an opportunity for future research.

Implementation of the 1,7-mRCTR intervention in three districts of Tanzania has demonstrated the potential of South-South cooperation initiatives in tackling global health challenges. This study and the previous pilot study are part of the International Forum on Surveillance-response System Leading to Tropical Diseases Elimination (ISRS) supported by National Institute of Parasitic Diseases, China CDC, the Swiss Tropical and Public Health Institute, and WHO. Bringing together policymakers, program administrators, frontline health workers, and researchers, the ISRS serves as a knowledge- and resource-sharing platform to promote cross-border cooperation in malaria elimination [31]. Through ISRS, China and Tanzania tailored China’s original “1–3–7” practices to suit Tanzania’s health system structure and malaria control priorities, activated inter-sectoral coordination mechanisms within the Tanzania government, leveraged funding from both international donors and China’s Belt and Road Initiative, and developed tools for diagnosis, treatment, and vector control based on China’s experience [32]. Although implementation was interrupted due to COVID-19, the established platform made it possible for the counterparts to share experiences and develop diagnosis and treatment guidelines for malaria and COVID-19 co-infection [32]. As China plays a growing role in global health governance and bilateral health development cooperation [33], the implementation of the 1,7-mRCTR approach in Tanzania serves as an example of overcoming common challenges in South-South cooperation, such as poor coordination, inadequate political commitment, language barriers, and inadequate financing, while contributing to the evidence on how to not only implement such initiatives but also how to monitor and evaluate their impacts [34].

The study has several limitations. First, since we only had household data from two cross-sectional surveys, we could not assess whether time trends in malaria prevalence were similar in intervention and control areas. We conducted several robustness checks to assess whether our results would change under different model specifications, but the effectiveness of the 1,7-mRCTR approach in moderate- to high-transmission settings could be further explored using a randomized controlled trial design. Second, the villages that received community tests and treatment were not always the highest-ranked villages based on the weekly incidence data. Reasons for this discrepancy include delay in uploading surveillance data from health facilities for the study team to generate the full ranking of all villages, a village having already been targeted recently, or weather and other logistical challenges. Although such implementation fidelity issues could be a concern, the relatively long implementation timeline and frequent updates of surveillance data allowed villages that had been missed to be targeted later on, reducing the concern that some villages with a high malaria burden would never have been targeted. Third, the DID method could not appropriately estimate the impact of the 1,7-mRCTR approach if other events affected malaria prevalence in the intervention and control areas differently. We ruled out several of these potential factors, and we are not aware of any other malaria programs during the intervention period. Fourth, this study relied on RDTs for malaria diagnosis. RDT limitations in detecting low parasitemia infections are well established [35, 36]. Although the study planned to use PCR and microscopy as a confirmative test and collected blood samples, we were not able to complete microscopic examination or compare PCR-confirmed infections that were missed by RDT due to the lack of resources. While RDTs are currently the primary diagnostic tool available to provide immediate results in the field, more sensitive diagnostics such as loop-mediated isothermal amplification would be more effective in detecting asymptomatic infections. Lastly, we did not track individuals who had not previously been tested in the follow-up rounds. While such tracking could increase testing coverage, it would also substantially raise the costs of the 1,7-mRCTR approach in our study setting.

## Conclusions

Combining elements of reactive “1–3–7” surveillance and targegted treatment strategies, the 1,7-mRCTR approach is a unique intervention that has demonstrated promising results in reducing malaria prevalence in moderate- to high-transmission areas of Tanzania. Investments in strengthening the routine surveillance infrastructure are essential to support the scale-up of new strategies to better identify malaria hotspots and target interventions at the village level. Importantly, this study demonstrated that Tanzania’s community-based healthcare worker network can be effectively leveraged to deliver a village-based reactive screen and treatment program to drive down malaria prevalence in hotspot areas. Furthermore, the 1,7-mRCTR project exemplifies opportunities for malaria-endemic countries to work together with countries that have been recently successful in eliminating malaria as part of effective South-South Cooperation initiatives.

### Supplementary Information

**Additional file 1:** **Table S1.** Comparison of key characteristics before and after matching. **Table S2.** Effects of 1,7-mRCTR on malaria prevalence and fever, probit models. **Table S3.** Comparison of regression models with different sets of controls. **Table S4.** Comparison of different clustering levels for standard errors. **Table S5.** Effects of 1,7-mRCTR by age groups. **Table S6.** Effects of 1,7-mRCTR by sex. **Table S7.** Effects of 1,7-mRCTR by household head’s education level. **Table S8.** Effects of 1,7-mRCTR by wealth index quintiles. **Table S9.** Changes in knowledge and travel history. **Table S10.** Changes in household characteristics. **Figure S11.** Changes in precipitation and temperature in study areas.

## Data Availability

The datasets used and/or analyzed during the current study are available from the corresponding author on reasonable request.

## Abbreviations

1,7-mRCTR: 1,7-Malaria Reactive Community-Based Testing and Response
RACD: Reactive case detection
CHCW: Community-based health care worker
MIR: Malaria incidence rate
RDT: Rapid diagnostic test
DID: Difference-in-differences
*CI*: Confidence interval
PCR: Polymerase Chain Reactions
ISRS: International Forum on Surveillance-response System Leading to Tropical Diseases Elimination
MSAT: Mass Screening and Treatment
