# Supplementary material for: Impact of 1,7-malaria reactive community-based testing and response (1,7-mRCTR) approach on malaria prevalence in Tanzania
Source: Infect Dis Poverty. 2023 Dec 18;12:116. doi: 10.1186/s40249-023-01166-0 (PMC10726614; doi:10.1186/s40249-023-01166-0)
Supplement: Supplementary file 1 — Additional file 1: Table S1. Comparison of key characteristics before and after matching. Table S2. Effects of 1,7-mRCTR on malaria prevalence and fever, probit models. Table S3. Comparison of regression models with different sets of controls. Table S4. Comparison of different clustering levels for standard errors. Table S5. Effects of 1,7-mRCTR by age groups. Table S6. Effects of 1,7-mRCTR by sex. Table S7. Effects of 1,7-mRCTR by household head’s education level. Table S8. Effects of 1,7-mRCTR by wealth index quintiles. Table S9. Changes in knowledge and travel history. Table S10. Changes in household characteristics. Figure S11. Changes in precipitation and temperature in study areas. [file 40249_2023_1166_MOESM1_ESM.docx]

# Additional information

Table S1. Comparison of key characteristics before and after matching

Table S2. Effects of 1,7-mRCTR on malaria prevalence and fever, probit models

Table S3. Comparison of regression models with different sets of controls

Table S4. Comparison of different clustering levels for standard errors

Table S5. Effects of 1,7-mRCTR by age groups

Table S6. Effects of 1,7-mRCTR by sex

Table S7. Effects of 1,7-mRCTR by household head’s education level

Table S8. Effects of 1,7-mRCTR by wealth index quintiles

Table S9. Changes in knowledge and travel history

Table S10. Changes in household characteristics

Figure S11. Changes in precipitation and temperature in study areas

Table S1. Comparison of key characteristics before and after coarsened exact matching

|  | *Villages in both baseline and endline* | | | *Villages retained using*  *Coarsened Exact Matching* | | |
| --- | --- | --- | --- | --- | --- | --- |
| **Variable** | **Comparison** | **Intervention** | ***P*-value** | **Comparison** | **Intervention** | ***P*-value** |
| *N* | 27 | 58 |  | 21 | 34 |  |
| Female | 0.62 | 0.62 | 0.76 | 0.62 | 0.61 | 0.52 |
| *Age* | 26.05 | 22.57 | < 0.001 | 24.88 | 24.39 | 0.41 |
| Primary school – member | .89 | .88 | 0.99 | 0.90 | 0.89 | 0.79 |
| Primary school – HH head | .65 | .61 | 0.13 | 0.66 | 0.63 | 0.35 |
| Slept under net last night | .84 | .88 | 0.062 | 0.85 | 0.87 | 0.37 |
| Flush toilet | 0.03 | 0.06 | 0.076 | 0.03 | 0.06 | 0.07 |
| Improved water source | 0.78 | 0.86 | 0.055 | 0.82 | 0.86 | 0.19 |
| Owns house | 0.91 | 0.86 | 0.12 | 0.92 | 0.89 | 0.13 |
| Has health insurance | 0.10 | 0.16 | 0.071 | 0.10 | 0.15 | 0.21 |
| Malaria prevalence | 0.23 | 0.28 | 0.15 | 0.25 | 0.28 | 0.55 |

* Descriptive statistics show village-level means based on data from the baseline survey.

The coarsened exact matching algorithm sorted all observations into strata, each of which has identical values for all the coarsened pre-treatment covariates, and then discarded all observations within any stratum that did not have at least one observation for each unique value of the treatment variable. This generates weights that could be applied to regular regression models. Since the household survey data was not a true panel (i.e., households/individuals interviewed at endline were different from those in the baseline), we used baseline data aggregated to the village level for the matching and dropped three villages that only appeared in the baseline or endline. We used two matching variables, age, and improved water source, and used the default binning algorithm. The algorithm retained 55 out of 85 villages after matching.

Table S2. Effects of 1,7-mRCTR on malaria prevalence and fever, probit models

|  | (1) | (2) |
| --- | --- | --- |
|  | All Districts | Rufiji |
| *Positive malaria test result, all age groups* | -0.046^***^ | -0.036^**^ |
|  | [-0.063, -0.029] | [-0.060, -0.012] |
| Observations | 24,094 | 4926 |
| Mean | 0.260 | 0.072 |
| Village | 86 | 22 |
| *Self-reported fever in past 14 days, under five years of age* | -0.031^**^ | 0.034 |
|  | [-0.051, -0.011] | [-0.018, 0.086] |
| Observations | 5211 | 1060 |
| Mean | 0.154 | 0.143 |
| Villages | 80 | 21 |

95% confidence intervals in brackets

Probit models show average marginal effects of the intervention, assuming all individuals in the sample were at the endline instead of the baseline.

Included village fixed effects and controlled for household characteristics (mosquito nets, flush toilet, improved source of drinking water, house ownership, health insurance) and individual member characteristics (age and sex).

The numbers of villages are different due to changes in administrative boundaries and missingness in reporting.

Standard errors clustered at the household level.

Data at household member level.

^*^ *P* < 0.05, ^**^ *P* < 0.01, ^***^ *P* < 0.001

Table S3. Comparison of regression models with different sets of controls

|  | (1) | (2) | (3) | (4) | (5) | (6) |
| --- | --- | --- | --- | --- | --- | --- |
| Intervention # Endline | -0.045^***^ | -0.045^***^ | -0.051^***^ | -0.050^***^ | -0.051^***^ | -0.045^***^ |
|  | [-0.067, -0.023] | [-0.067, -0.023] | [-0.074, -0.029] | [-0.072, -0.028] | [-0.073, -0.029] | [-0.067, -0.023] |
| Endline | -0.122^***^ | -0.122^***^ | -0.107^***^ | -0.106^***^ | -0.103^***^ | -0.122^***^ |
|  | [-0.139, -0.104] | [-0.139, -0.104] | [-0.124, -0.089] | [-0.123, -0.089] | [-0.120, -0.086] | [-0.139, -0.104] |
| Female | -0.036^***^ | -0.036^***^ |  |  |  | -0.036^***^ |
|  | [-0.047, -0.026] | [-0.047, -0.026] |  |  |  | [-0.047, -0.026] |
| 5 to 15 years | 0.088^***^ | 0.088^***^ |  |  |  | 0.088^***^ |
|  | [0.075, 0.101] | [0.075, 0.101] |  |  |  | [0.075, 0.101] |
| above 15 years | -0.082^***^ | -0.082^***^ |  |  |  | -0.082^***^ |
|  | [-0.094, -0.070] | [-0.094, -0.070] |  |  |  | [-0.094, -0.070] |
| Has mosquito nets | 0.007 | 0.007 | 0.008 |  |  | 0.007 |
|  | [-0.008, 0.021] | [-0.008, 0.021] | [-0.007, 0.023] |  |  | [-0.008, 0.021] |
| Has flush toilet | -0.028^**^ | -0.028^**^ | -0.032^**^ | -0.038^***^ |  | -0.028^**^ |
|  | [-0.047, -0.008] | [-0.047, -0.008] | [-0.052, -0.013] | [-0.058, -0.019] |  | [-0.047, -0.008] |
| Improved water | -0.014^*^ | -0.014^*^ | -0.016^*^ | -0.016^*^ |  | -0.014^*^ |
|  | [-0.028, -0.001] | [-0.028, -0.001] | [-0.030, -0.002] | [-0.030, -0.002] |  | [-0.028, -0.001] |
| Self-owned household | 0.024^**^ | 0.024^**^ | 0.027^***^ | 0.028^***^ | 0.032^***^ | 0.024^**^ |
|  | [0.009, 0.039] | [0.009, 0.039] | [0.012, 0.042] | [0.013, 0.043] | [0.017, 0.047] | [0.009, 0.039] |
| Owns health insurance | -0.038^***^ | -0.038^***^ | -0.035^***^ |  |  | -0.038^***^ |
|  | [-0.057, -0.018] | [-0.057, -0.018] | [-0.054, -0.015] |  |  | [-0.057, -0.018] |
| Constant | 0.373^***^ | 0.373^***^ | 0.338^***^ | 0.342^***^ | 0.322^***^ | 0.373^***^ |
|  | [0.328, 0.419] | [0.328, 0.419] | [0.293, 0.383] | [0.300, 0.384] | [0.281, 0.363] | [0.328, 0.419] |
| Observations | 24,102 | 24,102 | 24,102 | 24,102 | 24,102 | 24,102 |
| Adjusted *R*^2^ | 0.1537 | 0.1537 | 0.1153 | 0.1148 | 0.1142 | 0.1537 |
| Mean | 0.260 | 0.260 | 0.260 | 0.260 | 0.260 | 0.260 |
| Village | 88 | 88 | 88 | 88 | 88 | 88 |

95% confidence intervals in brackets

Included village fixed effects. Standard errors clustered at the household level.

Data at household member level.

^*^ *P* < 0.05, ^**^ *P* < 0.01, ^***^ *P* < 0.001

Table S4. Comparison of different clustering levels for standard errors, linear models

|  | (1) | (2) |
| --- | --- | --- |
|  | All districts | Rufiji |
| Cluster – household | -0.045^***^ | -0.046^**^ |
|  | [-0.067, -0.023] | [-0.081, -0.011] |
| Cluster – village | -0.045 | -0.046 |
|  | [-0.100, 0.009] | [-0.104, 0.012] |
| Observations | 24102 | 4926 |
| Mean | 0.260 | 0.072 |

Linear regression models

Coefficients and 95% confidence intervals (in brackets) for the interaction term “intervention * endline”.

Included village fixed effects and controlled for household characteristics (mosquito nets, flush toilet, improved source of drinking water, house ownership, health insurance) and individual member characteristics (age and sex).

The results in each row represent a different clustering indicated in the first column, and that each individual coefficient comes from a separate regression.

Data at household member level.

^*^ *P* < 0.05, ^**^ *P* < 0.01, ^***^ *P* < 0.001

Table S5. Effects of 1,7-mRCTR by age groups

|  | (1) | (2) |
| --- | --- | --- |
|  | All Districts | Rufiji |
| *Panel A: Below 5 years of age* |  |  |
| Intervention # Endline | -0.033 | -0.077^*^ |
|  | [-0.077, 0.012] | [-0.146, - 0.008] |
| Endline | -0.139^***^ | 0.037 |
|  | [-0.175, -0.103] | [-0.023, 0.097] |
| Observations | 5311 | 1093 |
| Adjusted *R*^2^ | 0.1594 | 0.0163 |
| Mean | 0.295 | 0.052 |
| *Panel B: 5 – 15 years of age* |  |  |
| Intervention # Endline | -0.016 | 0.019 |
|  | [-0.058, 0.026] | [-0.060, 0.099] |
| Endline | -0.204^***^ | -0.092^*^ |
|  | [-0.237, -0.170] | [-0.164, -0.020] |
| Observations | 7621 | 1556 |
| Adjusted *R*^2^ | 0.1925 | 0.0579 |
| Mean | 0.419 | 0.163 |
| *Panel C: Above 15 years of age* |  |  |
| Intervention # Endline | -0.056^***^ | -0.057^**^ |
|  | [-0.081, -0.031] | [-0.094, -0.021] |
| Endline | -0.068^***^ | 0.002 |
|  | [-0.087, -0.049] | [-0.029, 0.033] |
| Observations | 11,170 | 2277 |
| Adjusted *R*^2^ | 0.0738 | 0.0311 |
| Mean | 0.166 | 0.041 |

95% confidence intervals in brackets

Linear probability models included village fixed effects and controlled for household characteristics (mosquito nets, flush toilet, improved source of drinking water, house ownership, health insurance) and individual member characteristics (age and sex).

Standard errors clustered at the household level.

Data at household member level.

^*^ *P* < 0.05, ^**^ *P* < 0.01, ^***^ *P* < 0.001

Table S6. Effects of 1,7-mRCTR by sex

|  | (1) | (2) |
| --- | --- | --- |
|  | All districts | Rufiji |
| *Panel A: Male* |  |  |
| Intervention # Endline | -0.057^**^ | -0.041 |
|  | [-0.093, -0.021] | [-0.099, 0.018] |
| Endline | -0.132^***^ | -0.024 |
|  | [-0.160, -0.104] | [-0.075, 0.027] |
| Observations | 9258 | 1844 |
| Adjusted *R*^2^ | 0.1643 | 0.0373 |
| Mean | 0.304 | 0.089 |
| *Panel B: Female* |  |  |
| Intervention # Endline | -0.038^**^ | -0.051^*^ |
|  | [-0.064, -0.012] | [-0.090, -0.011] |
| Endline | -0.116^***^ | -0.006 |
|  | [-0.137, -0.096] | [-0.041, 0.029] |
| Observations | 14,844 | 3082 |
| Adjusted *R*^2^ | 0.1385 | 0.0359 |
| Mean | 0.232 | 0.061 |

95% confidence intervals in brackets

Included village fixed effects and controlled for household characteristics (mosquito nets, flush toilet, improved source of drinking water, house ownership, health insurance) and individual member characteristics (age).

Standard errors clustered at the household level.

Data at household member level.

Table S7. Effects of 1,7-mRCTR by household head’s education level

|  | (1) | (2) |
| --- | --- | --- |
|  | All districts | Rufiji |
| *Panel A: : Household head did not complete primary school* |  |  |
| Intervention # Endline | -0.039 | 0.030 |
|  | [-0.082, 0.004] | [-0.031, 0.090] |
| Endline | -0.137^***^ | -0.085^**^ |
|  | [-0.171, -0.103] | [-0.137, -0.033] |
| Observations | 6876 | 1390 |
| Adjusted *R*^2^ | 0.1713 | 0.0646 |
| Mean | 0.251 | 0.088 |
| *Panel B: Household head completed primary school* |  |  |
| Intervention # Endline | -0.038^**^ | -0.061^**^ |
|  | [-0.064, -0.012] | [-0.101, -0.021] |
| Endline | -0.116^***^ | 0.004 |
|  | [-0.136, -0.095] | [-0.031, 0.040] |
| Observations | 17,226 | 3536 |
| Adjusted *R*^2^ | 0.1449 | 0.0323 |
| Mean | 0.263 | 0.065 |

95% confidence intervals in brackets

Included village fixed effects and controlled for household characteristics (mosquito nets, flush toilet, improved source of drinking water, house ownership, health insurance) and individual member characteristics (age and sex).

Standard errors clustered at the household level.

Data at household member level.

^*^ *P* < 0.05, ^**^ *P* < 0.01, ^***^ *P* < 0.001

Table S8. Effects of 1,7-mRCTR by wealth index quintiles

|  | (1) | (2) | (3) | (4) | (5) |
| --- | --- | --- | --- | --- | --- |
|  | Lowest | Lower | Middle | Higher | Highest |
| Intervention # Endline | -0.063^*^ | -0.081^**^ | -0.054^*^ | -0.039 | -0.038 |
|  | [-0.116, -0.010] | [-0.131, -0.030] | [-0.100, -0.007] | [-0.091, 0.012] | [-0.086, 0.011] |
| Endline | -0.161^***^ | -0.115^***^ | -0.131^***^ | -0.131^***^ | -0.047^*^ |
|  | [-0.199, -0.122] | [-0.154, -0.075] | [-0.165, -0.096] | [-0.173, -0.090] | [-0.089, -0.005] |
| Observations | 4652 | 4926 | 4932 | 4676 | 4916 |
| Adjusted *R*^2^ | 0.1698 | 0.1474 | 0.1710 | 0.1655 | 0.1265 |
| Mean | 0.318 | 0.266 | 0.250 | 0.257 | 0.177 |

95% confidence intervals in brackets

Wealth index is constructed using principal component analysis based on the following household assets: iron/tile roof, window protected from mosquito entry, electricity/solar as light source, flush toilet, improved water source, house ownership, ownership of household items, and ownership of means of transportation.

Included village fixed effects and controlled for household characteristics (mosquito nets, flush toilet, improved source of drinking water, house ownership, health insurance) and individual member characteristics (age and sex).

Standard errors clustered at the household level.

Data at household member level.

^*^ *P* < 0.05, ^**^ *P* < 0.01, ^***^ *P* < 0.001

Table S9. Changes in knowledge and travel history

|  | (1) | (2) | (3) | (4) | (5) |  |
| --- | --- | --- | --- | --- | --- | --- |
|  | Know what malaria is | Number of malaria symptoms listed | Number of malaria prevention measures listed | Travelled outside of village | Number of travel days outside of village |  |
| Intervention # Endline | 0.005 | -0.140^***^ | -0.029 | -0.020^***^ | -1.973 |  |
|  | [-0.025, 0.035] | [-0.215, -0.065] | [-0.091, 0.033] | [-0.031, -0.008] | [-6.881, 2.936] |  |
| Endline | 0.049^***^ | 0.158^***^ | 0.197^***^ | -0.035^***^ | -2.194 |  |
|  | [0.026, 0.072] | [0.100, 0.216] | [0.151, 0.243] | [-0.044, -0.026] | [-4.618, 0.229] |  |
| Observations | 14,751 | 11,212 | 11,211 | 24,061 | 880 |  |
| Adjusted *R*^2^ | 0.0962 | 0.0418 | 0.0529 | 0.0312 | 0.0404 |  |
| Mean | 0.741 | 1.839 | 1.406 | 0.056 | 9.752 |  |

95% confidence intervals in brackets

Knowledge questions were only asked if the respondent was above 5 years of age.

Malaria symptoms included chills, fever, cold, and, headache.

Malaria prevention measures included environmental hygiene and cleanliness, mosquito net use, and mosquito repellent/incense.

Included village fixed effects and controlled for household characteristics (mosquito nets, flush toilet, improved source of drinking water, house ownership, health insurance) and individual member characteristics (age and sex).

Standard errors clustered at the household level.

Data at household member level.

^*^ *P* < 0.05, ^**^ *P* < 0.01, ^***^ *P* < 0.001

Table S10. Changes in household characteristics

|  | (1) | (2) | (3) | (4) | (5) | (6) | (7) | (8) | (9) | (10) |
| --- | --- | --- | --- | --- | --- | --- | --- | --- | --- | --- |
|  | Female household head | Household head completed primary school | Owns house | Land | Improved source of drinking water | Flush toilet | Health insurance | Has any treated mosquito net | Agriculture as main income source | Number of household members |
| Intervention # Endline | 0.035 | 0.040 | -0.000 | -0.496 | 0.082 | -0.018 | -0.033 | 0.099 | 0.019 | -0.362^*^ |
|  | [-0.011, 0.082] | [-0.010, 0.090] | [-0.029, 0.029] | [-1.041, 0.049] | [-0.011, 0.176] | [-0.043, 0.006] | [-0.074, 0.007] | [-0.004, 0.201] | [-0.020, 0.058] | [-0.655, -0.069] |
| Endline | 0.024 | 0.090^***^ | 0.024^*^ | 0.152 | -0.167^***^ | -0.007 | -0.047^**^ | -0.129^**^ | 0.047^**^ | 0.088 |
|  | [-0.012 ,0.060] | [0.052, 0.128] | [0.006, 0.043] | [-0.328, 0.633] | [-0.247, -0.086] | [-0.021, 0.007] | [-0.076, -0.018] | [-0.213, -0.044] | [0.019, 0.075] | [-0.117, 0.292] |
| Observations | 11,235 | 11,235 | 11,235 | 10,834 | 11,235 | 11,235 | 11,235 | 11,235 | 11,235 | 10,834 |
| Adjusted *R*^2^ | 0.0150 | 0.0436 | 0.0436 | 0.0590 | 0.1402 | 0.0421 | 0.0698 | 0.0431 | 0.0457 | 0.0367 |
| Mean | 0.275 | 0.688 | 0.903 | 4.183 | 0.778 | 0.044 | 0.093 | 0.652 | 0.866 | 4.454 |

95% confidence intervals in brackets

Included village fixed effects.

Standard errors clustered at the village level.

Data at household level.

^*^ *P* < 0.05, ^**^ *P* < 0.01, ^***^ *P* < 0.001

Figure S11. Changes in precipitation and temperature in study areas

Trend of weekly averaged precipitation and temperature in treatment (T) and control (C) wards. Red lines indicate the week of baseline (July 24–September 4, 2019) and endline (September 20–October 27, 2021).
